# Bone graft from greater trochanter in posterior wall fractures with impacted fragments

**DOI:** 10.1007/s10195-014-0291-1

**Published:** 2014-03-27

**Authors:** R. Pascarella, M. Commessatti, R. Politano, A. Maresca, M. Del Torto, M. Chehrassan, R. Fantasia

**Affiliations:** 1 Orthopedic and Traumatology Department, Riuniti Hospital, Ancona, Italy; 2 Orthopedic and Traumatology Department, Maggiore Hospital, Bologna, Italy; 3 Orthopedic and Traumatology Department, Rizzoli Orthopaedics Institute, Bologna, Italy; 4 Ospedali Riuniti, via Conca n.71, 60126 Torrette, Ancona, Italy

**Keywords:** Posterior wall fractures, Impacted fragment, Bone loss, Autograft, Hip dislocation, Intra-articular fragments

## Abstract

**Background:**

Posterior wall fracture is the most common acetabular fracture. Comminuted fractures with an impacted segment represent a subtype of this injury. The subchondral bone of the articular zone is compressed and causes a bone defect. The impacted fragment should be isolated, mobilized, and then reduced. A bone graft should be used to fill the gap. The other fragments are fixed following the reduction of the impacted segment.

**Materials and methods:**

Ten patients with comminuted fractures and impacted segments with bone defects were enrolled in our study, from January 2010 to July 2012. Autogenous bone grafts from the greater trochanter were used to fill the gap in all patients. The reduction was achieved through the insertion of the graft above the impacted fracture, and plate fixation was performed subsequently. Merle d’Aubigne and Postel scoring, modified by Matta, was applied to evaluate the patients during follow-up. The mean follow-up was 12 months.

**Results:**

The clinical results included one “excellent”, four “very good”, four “good” and one “poor”. Pain in the zone of graft harvesting was not detected in any patient. Femoral head necrosis was observed in one case. No other severe complications were detected.

**Conclusions:**

Using an autogenous bone graft to fill the bone defect supplies excellent mechanical stability without any severe complications at the donor site. This surgical technique seems to be effective and safe in treating a comminuted fracture of the posterior wall in association with an impacted segment.

**Level of evidence:**

Level IV.

## Introduction

Posterior acetabular wall fracture is the most common type of acetabular fracture [1–9], accounting for 47 % of total acetabular fractures, according to Letournel and Judet [4]. The majority of posterior wall fractures are comminuted with impacted fragments [10–12], usually in the elderly population. This fracture usually occurs in association with posterior hip dislocation, which leads to displacement of bone fragments [2, 13, 14]. According to the Judet classification, this fracture can be divided into two types. The first type includes free fragments or fragments attached to the joint capsule. The second type includes impacted fragments, with or without bone loss [4]. Comminuted fractures are usually seen in females over 50 years old and in elderly populations due to osteoporosis which increases bone fragility [15–17]. The reconstruction of the posterior wall is technically demanding. This can be more complicated when more than 50 % of the joint surface is involved, which may lead to hip joint instability [1, 6, 11, 12]. Many studies also emphasize the importance of the surgeon’s experience; it has been demonstrated that 19–25 % of fair or poor results may occur following surgeries performed by experienced surgeons, whereas this may increase to 55–56 % when the surgery is performed by less experienced surgeons [4, 18–20]. The aim of this study was to assess the results of the surgical technique for the treatment of comminuted posterior acetabular wall fracture in association with an impacted segment using an autogenous trochanteric bone graft.

## Materials and methods

**Fig. 1:**
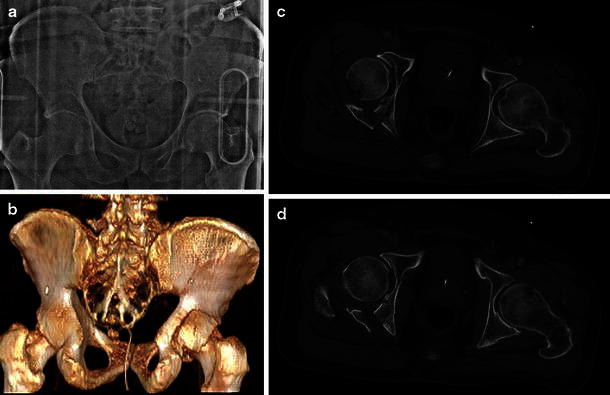
Preoperative **a** X-ray, **b–d** 3-D and 2-D CT scans (Male, 73 years, car accident)

Twenty-six patients with posterior wall fractures were operated on in our center from January 2010 to July 2012. Out of these, ten patients, including nine males and one female, were enrolled in our study with the inclusion criteria of comminuted posterior acetabular wall fractures and impacted segments with bone defects. The mean age was 57.6 (range 26–89 years). According to our trauma protocol, all the patients were evaluated clinically and radiographically preoperatively. Imaging studies included AP and Judet oblique views and 2- and 3-D CT scans of the pelvis [2, 4, 15] (Fig. 1). All cases were treated surgically by two experienced surgeons (RP & MC). The mechanism of injuries were as follows: seven car accidents, one motorcycle accident, one fall from height, and one fall from a chair. Posterior hip dislocation was observed in five patients while free fragments were detected in three of them.

In four cases hip dislocation was reduced within 6 h of injury; in one case a close reduction failed and an open reduction was performed at the time of surgery, after 5 days when the patient’s general condition permitted surgery (Table 1).

**Table 1:** Patient’s demographic and fracture characteristics

Case	Age	Sex	Mechanism of injury	Hip dislocation	Time of hip reduction	Intra-articular fragment	Other complication
I	59	M	Car accident	No		No	
II	89	M	Fall from height	Yes	5 Days after trauma	No	Osteonecrosis
III	26	M	Car accident	No		No	
IV	70	M	Car accident	No		No	
V	81	M	Fall from chair	No		No	
VI	45	M	Car accident	Yes	Within 6 h of injury	Yes	
VII	50	F	Car accident	Yes	Within 6 h of injury	No	
VIII	73	M	Car accident	Yes	Within 6 h of injury	No	
IX	56	M	Car accident	Yes	Within 6 h of injury	Yes	
X	27	M	Motocycle accident	No	Within 6 h of injury	Yes	

**Fig. 2:**
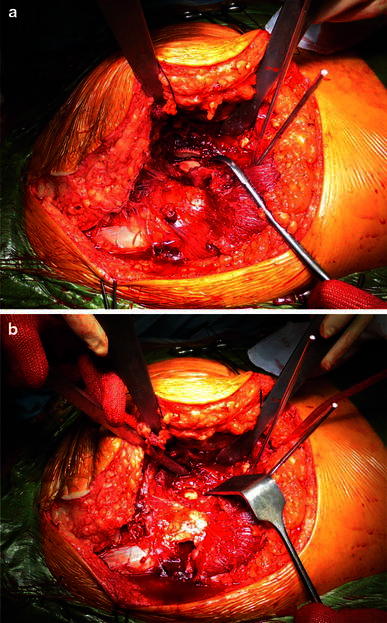
**a, b** Impacted fragment and lack of bone substance; femoral head as a point of reference

**Fig. 3:**
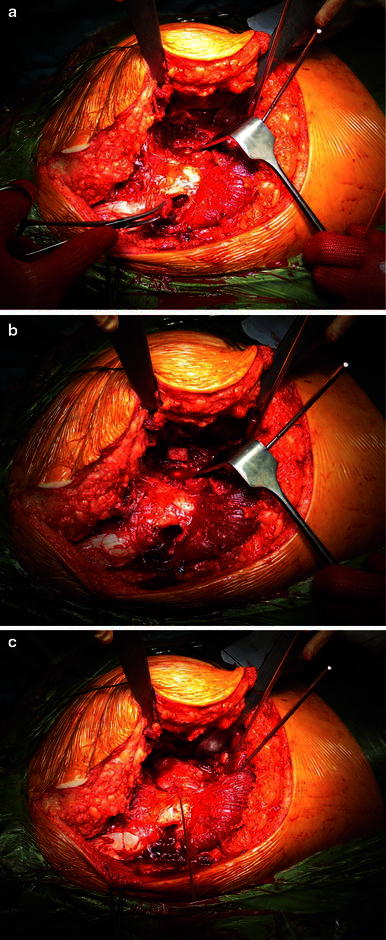
**a–c** Cancellous bone graft from greater trochanter to fill the gap, after reduction of articular fragment

**Fig. 4:**
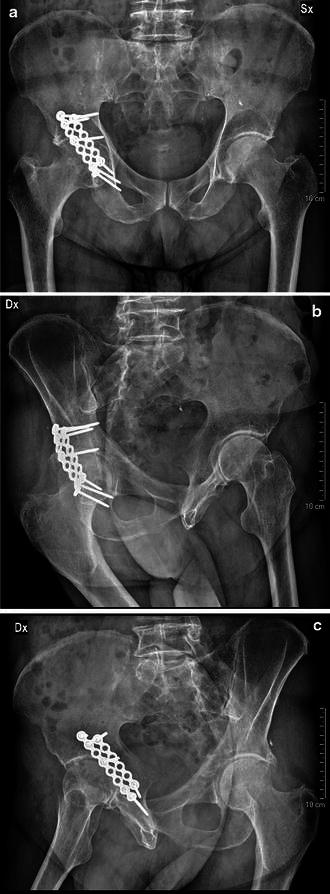
Post-operative X-rays: **a** AP and **b, c** oblique Judet views (same patient as in Fig. 1)

A Kocher–Langenbeck approach was used for all patients [2, 4, 21]. Patients were placed in the prone position. The knee was flexed to minimize the chance of sciatic nerve injury. After detaching the piriformis tendon and conjoined tendons, including obturator internus and gemelli muscles, the greater sciatic notch, the ischial spine and the lesser sciatic notch were exposed. Two retractors were inserted in the greater and the lesser sciatic notches to expose the posterior column in its whole extent. The femoral head was re-dislocated in the case of intra-articular fragments and articular lavage was performed. While the fracture was isolated, the hematoma was evacuated and the existing fragments were identified. The femoral head was used as a landmark to guide the surgeons when reducing the fragments. A 2 cm × 2 cm bone graft from the greater trochanter was harvested and inserted into the identified bone defect (Fig. 2); the bone graft was placed over the reduced fragment to hold it in place (Fig. 3). The size of the bone graft should be proper for the bone defect; otherwise the reduction will not be anatomically correct. Definitive fixation was finally performed using one or two plates (Fig. 4). The bone defect at the graft harvesting zone on the greater trochanter was covered by reattaching of the periosteal flaps.

**Fig. 5:**
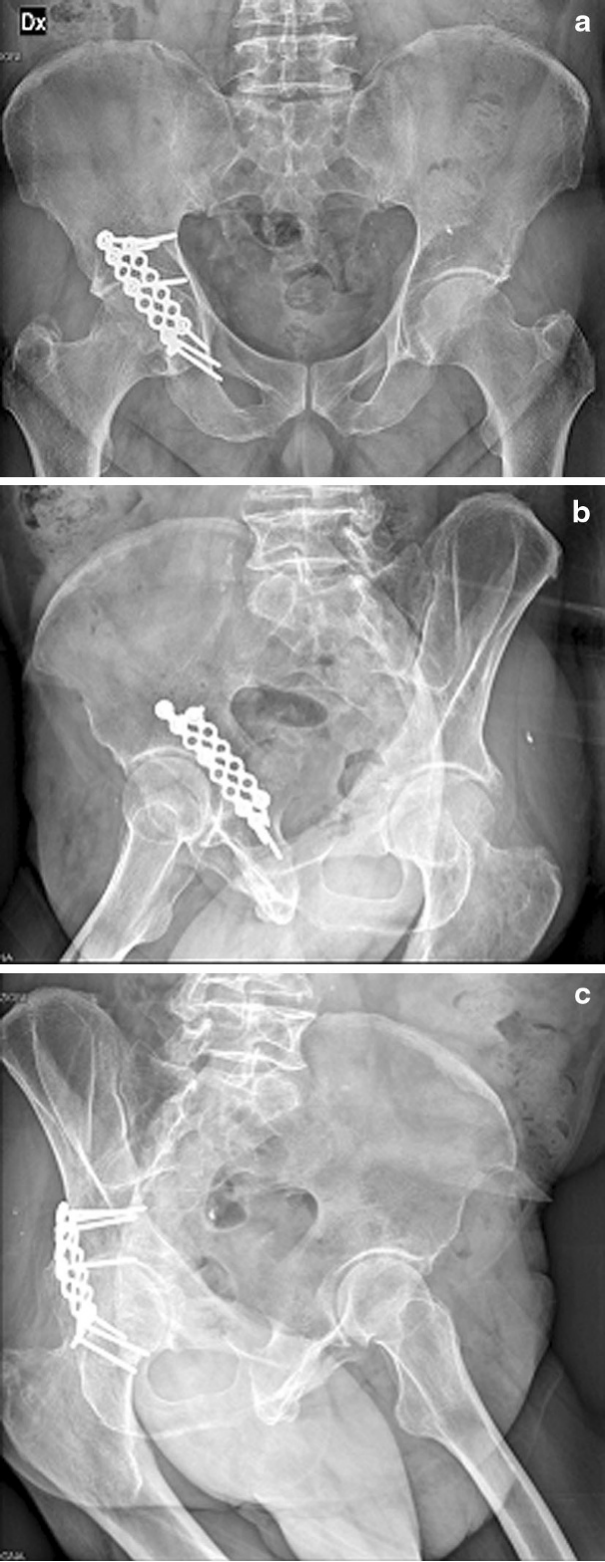
X-rays at the last follow-up of the same patient as in Fig. 1 (May, 2013): **a** AP and **b, c** oblique Judet views

The post-operative rehabilitation protocol included immediate passive and active flexion–extension of the hip with no weight-bearing for 12 weeks. All patients were followed clinically and radiographically after 1, 3, 6 and 12 months following surgery (Fig. 5). The patients were evaluated clinically using Merle d’Aubigne and Postel scoring modified by Matta. According to this clinical score system, pain, gait and range of motion of the hip have a maximum of six points and the final score is the sum of the three values [4, 22–24]. The radiographic evaluation was performed using the radiologic criteria of Matta [22].

## Results

The clinical results according to Merle d’Aubigne and Postel scoring modified by Matta were as follows: excellent (=18 points) in one case, very good (=17 points) in four cases, good (15–16 points) in four cases, poor (<13 points) in one case.

At the last follow-up all surgically treated fractures had been reduced anatomically. The radiologic grading at the last follow up was excellent (normal hip joint) in five cases and good (minimal sclerosis and joint space narrowing <1 mm) in four cases; in an 89-year-old male, due to an unstable general condition, the reduction of the femoral head was postponed for 5 days and femoral head necrosis was detected at the last follow-up with a poor radiological result (Tables 2, 3). There were no other severe complications. Pain in the zone of graft harvesting was not detected in any patient.

**Table 2:** Merle d’Aubigne and Postel scoring modified by Matta [4, 22–24]

	Number of patients
Excellent (=18 points)	1
Very good (=17 points)	4
Good (=15–16 points)	4
Poor (<13 points)	1

**Table 3:** Radiologic criteria of Matta [22]

	Number of patients
Excellent (normal hip joint)	5
Good (joint narrowing less than 1 mm)	4
Poor (advanced joint change)	1

## Discussion

Comminuted fracture in association with an impacted segment of the posterior wall occurs following femoral head dislocation, or when it sinks into the acetabulum, causing compression of the trabecular bone and consequent bone loss. A CT scan is indicated in any cases of acetabular fracture or hip dislocation. Due to inefficiency of plain X-ray alone to recognize the impacted segment or intra-articular fragments, a CT scan must be performed to provide a more accurate assessment of the fracture pattern.

The patient should be treated surgically within 7–10 days following the trauma, in order to get a good reduction. After 10 days, fibrous callus formation may make the surgical reduction less effective. In addition, early consolidation of impacted fragments can occur and may lead to a misdiagnosis of this type of fracture, which can result in malunion.

After the reduction of the fragments, different materials may be used to fill the bone defect, including artificial bone substitutes and allograft or autogenous cancellous bone grafts.

It is important to consider the mechanical properties of the material that is used to fill the gap. Inability of the substituted material to provide good mechanical properties leads to collapse of the graft following weight-bearing. This may result in impacted fragment reduction failure and nullifies the benefits of surgery. From a mechanical point of view, artificial or synthetic bone substitutes possess good osteointegrative and conductive properties; however, being completely reliant on viable periosteum/bone and the higher costs with respect to other options limits the use of artificial bone substitute [25–27]. The frozen allograft also provides good mechanical and biological properties, although the risk of infection and disease transmission remain the main concerns when using these grafts [28, 29]. An autogenous graft has by far the most osteogenic potential and in our opinion is the best choice for filling a bone defect in cases of comminuted fractures in association with an impacted segment. The autogenous graft may be harvested from the iliac crest near to the posterior superior iliac spine [30, 31] or from the greater trochanter. A second incision is required to take the graft from the iliac crest, which may add other complications such as irritation of the donor site in the following months [32–35]. Harvesting the graft from the greater trochanter does not need another surgical incision, and in our experience the graft provides good quality properties without resulting in any severe complications or donor-site pain. In our series we did not encounter any notable complications related to this surgical technique. However, femoral head necrosis was observed in one case due to non-reducible posterior hip dislocation in an 89-year-old patient without any relation to surgical technique. The best choice for the diagnosis of femoral head necrosis may be MRI, but the presence of metallic implants (plate) near to the hip joint can cause substantial image artifacts in MRI which make the diagnosis of femoral head necrosis very difficult or even impossible. We made the diagnosis using plain X-ray and CT scan. This surgical technique which uses trochanteric autogenous bone grafts provides good functionally and radiographically results. We believe that this technique can be safe and has a low risk of severe complications for the treatment of posterior acetabular wall fracture with impacted segments and bone defects. However, this study was clearly limited due to the small number of cases and the absence of a control group. The efficacy of this surgical technique needs a study with a longer follow-up to demonstrate osteoarthritic changes of the hip joint following this procedure.
